# Injuries of the Wrist Joint

**Published:** 1906-01-27

**Authors:** 


### INJURIES OF THE WRIST JOINT.

The difficulty of exact diagnosis of the nature of the injury when the wrist has been "sprained," has led to the subject receiving very little attention in text-books. In fact, until the $x$-rays could be applied, it was often impossible to recognise the altered relationships of the small bones of the carpus. In the majority of text-books it is stated that the most commonly displaced carpal bone is the os magnum and that the displacement is backwards. Codman [1] and Chase make a careful review of the literature of the subject and detail a number of cases which have come under their own observation and which have been submitted to the $x$-rays. From this they conclude that two lesions are of much greater frequency and importance than all others—namely, fracture of the scaphoid with or without dislocation of the proximal fragment, and anterior dislocation of the semilunar bone. The two injuries are often combined, in which case the proximal fragment of the scaphoid is dislocated forward with the semilunar. In the majority of cases the nature of the injury is not recognised until some time after the accident, and under these circumstances treatment is often difficult and may require an open operation. Most of the patients are men between twenty and forty, and the violence which gives rise to the injury is of an indirect nature and of great force, generally a fall from a height on the outstretched hand. Simple fracture of the scaphoid gives the following signs: Localised swelling on the radial side of the wrist, acute tenderness in the "anatomical snuff-box" when the hand is adducted, the limitation of extension of the wrist by muscular spasm, the overcoming of which causes unbearable pain, and a characteristic skiagram. A broken scaphoid has but little power of repair and gives rise to little or no callus. Fractures of the scaphoid which remain untreated or are treated by active and passive movement and massage, generally, if not always, remain ununited, and the original symptoms often persist for years with but slightly abated intensity. If, however, the hand and wrist be maintained in plaster of Paris for four or five weeks, the bone may unite. In cases in which an ununited fracture of the scaphoid gives rise to persistent symptoms, an incision is made along the back of the wrist parallel to, and to the outer side of the tendons of the common extensor. Through this the proximal half of the scaphoid can easily be removed. Passive movements of the wrist and active movements of the fingers are begun within a week of the operation. Anterior dislocation of the semilunar bone can often be diagnosed even without the *x*-ray. It is brought about by violent hyper-extension of the wrist, which forces the bone out from the interval between the os magnum and the radius. Groves [2] points out that the shape of the bone and the fact that its dorsal surface is much smaller than its palmar, causes it to be forced out anteriorly when the knuckles are violently driven against the radius. A "silver fork" deformity is produced very similar to that of Colles' fracture, but the diagnosis from this is readily made by noting that the styloid processes maintain unaltered relations. The posterior prominence of the deformity consists of the head of the os magnum, and it is this appearance which is responsible for the statement that posterior dislocation of the os magnum is the commonest carpal displacement. A swelling under the flexor tendons of the wrist is caused by the displaced semilunar. The palm is shortened as compared with the other hand, and the fingers are held stiff and flexed. Recent dislocations of the semilunar may be reduced with good result even after the fifth week, by hyper-extension, followed by hyperflexion of the wrist, counter presure being made by the tips of the thumb over the semilunar. Irreducible dislocations cause great pain from pressure on the branches of the median nerve. In such cases it is best to excise the bone through an anterior incision. In conclusion it may be stated that no carpal bone is exempt from injury, but that the cuneiform bone, owing probably to the play of movement allowed by its attachment to the triangular fibro-cartilage, is most rarely affected.

[1] Annals of Surg., June 1905. [2] Lancet, Aug. 13, 1904.

